# Enhancing Early Medical Education Through Patient Engagement: Creation of a Toolkit Informed by Experts by Experience

**DOI:** 10.1111/tct.70328

**Published:** 2025-12-18

**Authors:** Jaimy Saif, David Rogers, Claire Stocker

**Affiliations:** ^1^ Aston Medical School, College of Health and Life Sciences Aston University Birmingham UK; ^2^ Expert by Experience Birmingham UK

## Abstract

Incorporating the patient voice into health professional education enhances empathy, promotes person‐centred care and enriches learning. This cocreation article describes the development of a practical, feedback‐informed toolkit to support early medical education through expert by experience (EBE) engagement. EBEs from The Silverlining Brain Injury Charity contributed to the design via a qualitative study using open‐ended questionnaires. Thematic analysis identified six key themes: the importance of respectful engagement, logistical challenges, clarity of session expectations, recognition of EBE expertise, personal benefits of participation and ethical concerns. EBEs emphasised the need for dignity, structured facilitation, emotional safeguards and flexible delivery methods. The resulting toolkit is mapped directly to these themes. It includes guidance on planning, facilitation, ethical considerations, orientation and evaluation. Designed for Level 3 of the patient engagement spectrum, where EBEs share lived experiences in faculty‐facilitated teaching, the toolkit promotes meaningful, sustainable involvement. It responds to growing calls for coproduction in health education and serves as a replicable model for integrating patient insights into curriculum design and delivery. While based on a small, specialised sample, the depth and clarity of EBE feedback offer strong foundations for this resource. Future work should explore its adaptability across different healthcare disciplines and settings. To increase accessibility, the toolkit is available in two formats: as a shareable webpage and a navigable PDF document. This approach enables wider reach and sustained use by educators, ensuring that patient voices remain central to shaping future health professionals.

## 1 Introduction

There is growing recognition that meaningful patient involvement enriches health professional education and fosters empathetic, patient‐centred practice [1]. This article explores how expert by experience (EBE) feedback informed the development of a toolkit that promotes EBE engagement in early medical education. Toolkit approaches are increasingly used in healthcare education to embed complex themes like patient involvement into curricula [2]. This project focused on Level 3 of the patient engagement ladder [3] where EBEs share their lived experiences in faculty‐led teaching. EBEs from The Silverlining Brain Injury Charity contributed to the design, ensuring that the toolkit reflects authentic patient perspectives. Structured programmes such as the Expert Patient Programme have established formal roles for patient educators, particularly in chronic disease management [4, 5]. This study describes a distinct model of involvement in early medical education, where EBEs contribute through lived experience and prior teaching. It offers a practical toolkit to support educators initiating such engagement.

*This article explores how Expert by experience (EBE) feedback informed the development of a toolkit that promotes EBE engagement in early medical education*.

## 2 Study Design and Participants

A qualitative design was used to examine EBE experiences, motivations, expectations and concerns [6]. The study was designed with a narrow aim focused on EBE session design. Using the concept of information power, sample size was calculated based on three parameters: aim specificity, participant relevance and analytic strategy [7]. The aim was narrow; all participants had direct experience with the topic, and the analysis was structured to identify recurring patterns across responses. Based on these design parameters, six EBEs from The Silverlining Brain Injury Charity were invited to complete an anonymised questionnaire with eight open‐ended questions.

## 3 Data Collection and Analysis

Responses were analysed using inductive thematic analysis [8]. Each response was independently coded by two researchers. Coding decisions were reviewed in structured team discussion to resolve discrepancies and confirm category boundaries. Rigour was ensured through investigator triangulation, cross‐checking of interpretations and full‐team validation of themes against the dataset. Table 1 presents the final themes with supporting quotations, allowing traceability between raw data and analytic categories.

**TABLE 1:** A structured questionnaire was developed to explore the EBEs' perceptions and experiences comprehensively.

#	Question
1	How do EBEs perceive the opportunity to engage with EBE sessions?
2	What factors influence EBEs' decisions to participate in EBE sessions?
3	What expectations do EBEs have regarding their involvement in EBE sessions?
4	How do EBEs envision their role within EBE sessions?
5	What potential benefits do EBEs anticipate from participating in EBE sessions?
6	What concerns or reservations do EBEs express about engaging with EBE sessions?
7	How do EBEs believe their involvement in EBE sessions could impact their own healthcare journey?
8	What recommendations do EBEs have for improving the design and implementation of EBE sessions?

*Note:* The questionnaire consisted of eight open‐ended questions.

### 3.1 Ethical Considerations

Ethical approval was obtained from the HLS Ethics Committee. Participation was voluntary, anonymised and conducted with informed consent.

### 3.2 Themes Derived From EBE Feedback

**FIGURE 1:**
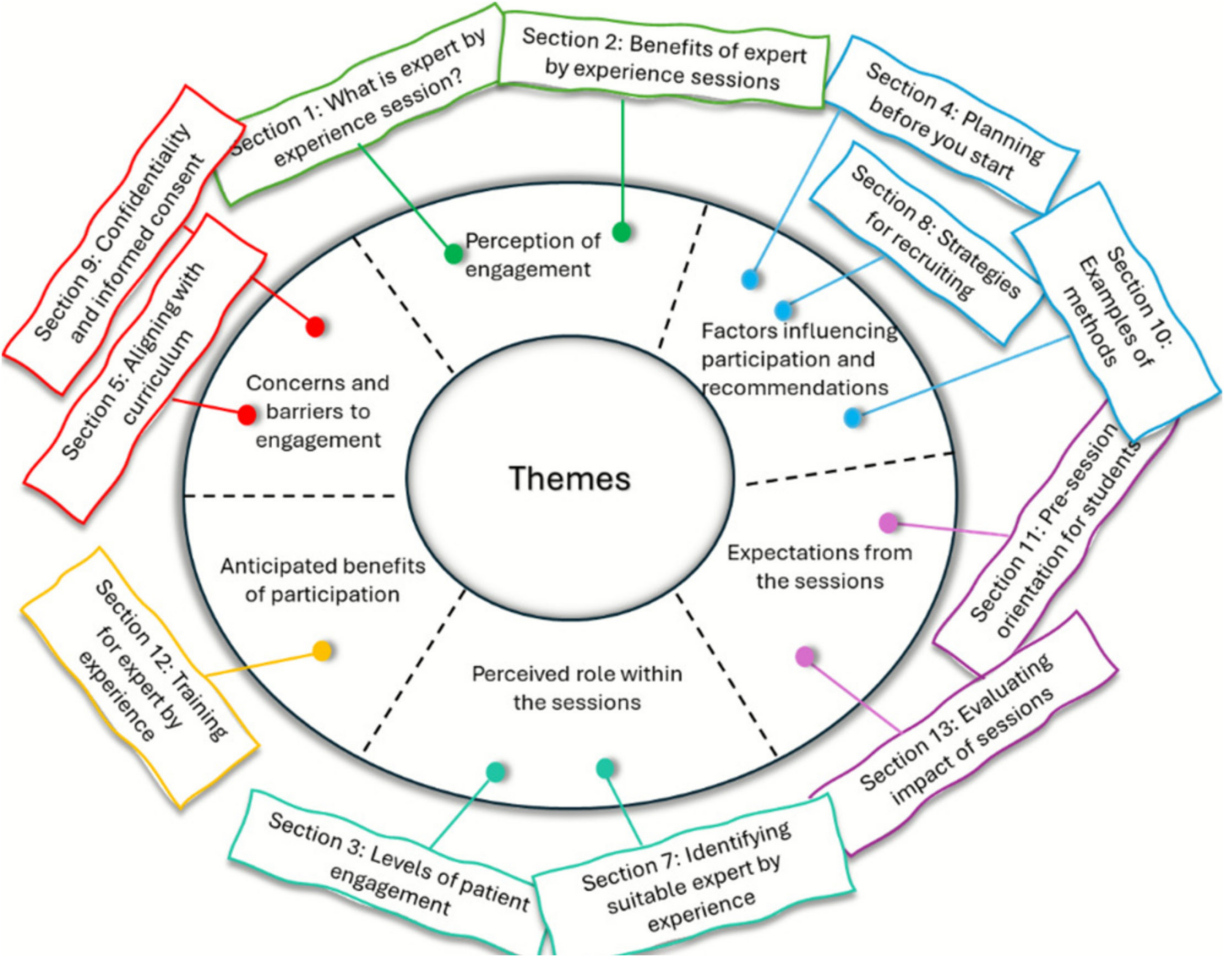
Themes developed from the EBE feedback and how the themes informed the contents of the toolkit.

EBE responses were analysed using inductive thematic analysis, following the six‐phase approach described by Braun and Clarke [8]. Two researchers independently coded the full dataset, then met to reconcile discrepancies and develop a preliminary theme structure. This structure was reviewed by the wider team to ensure consistency and alignment with the original data. Figure 1 presents the final themes, organised into categories that reflect barriers and enablers to participation in medical education sessions. These categories informed the structure and content of the toolkit.

### 3.3 Elements of the Toolkit

The development of this toolkit was driven by an iterative and collaborative process, integrating feedback directly from EBE participants to ensure it reflects the realities and needs of patients involved. Each section of the toolkit corresponds to themes derived from EBE input, highlighting the cocreation ethos underpinning its design. The toolkit is intended as a practical resource for educators considering the integration of EBE sessions into medical curricula. It begins with a clear rationale (‘Why this toolkit’) that outlines its aims, theoretical foundations and intended audience. A concise contents list provides an overview, enabling users to navigate to relevant sections efficiently. The ‘Before you begin’ session systematically guides educators through preparatory steps, allowing them to engage with the toolkit according to the stage of planning they are at.

*Each section of the toolkit corresponds to themes derived from EBE input, highlighting the cocreation ethos underpinning its design*.

### 3.4 Sections 1 and  2: The Importance of EBE Sessions

One of the central themes emerging from EBE feedback concerned perceptions of engagement and respect. EBEs emphasised that they wished to be recognised as individuals with valuable lived experience rather than treated merely as case studies (Table 2, EBE comment 1). This insight directly informed the ethos of the toolkit, particularly Section 1, which positions EBEs as critical contributors who bridge theoretical knowledge and lived experience. Section 2 further articulates the ways EBEs foster empathy and enrich clinical education, complementing existing literature that highlights potential emotional risks of patient participation in teaching [9, 10, 11]. These sections provide evidence of positive outcomes from EBE involvement, drawing on both student and patient feedback. The insights underscore the importance of cultivating respectful, supportive environments that acknowledge and safeguard patient contributions while enhancing learning.

**TABLE 2:** Comments from the EBEs are listed.

EBE	Comments
EBE comment 1:	‘It gave us the patients an opportunity to show others even though we have had a life‐threatening brain injury and survived we are still humans who joke and laugh but can communicate clearly in various in our own ways therefore we wish to be treated like ones. Hopefully showing the students they should treat their new patients with the same respect and dignity and not just another patient in the hospital bed.’
EBE comment 2:	‘From an EBE's perspective, the more sessions they participate in, the more repetitive and boring retelling their stories may become.’
EBE comment 3:	‘Produce brief videos of the EBEs telling their stories which could be played to the students in advance of a live Q&A session. This would avoid the need for the EBEs to have to repeat their stories a number of times but also provide the opportunity for the tutors and students to do some prework and encourage more participative Q&A sessions’.
EBE comment 4:	‘Well managed and facilitated sessions require facilitators who are able to draw out the relevant points of the EBEs’ stories for the students and help focus the questioning to develop these further. Without good facilitation, these sessions do not work anywhere near so well, EBEs can become disillusioned and you would question the value of the sessions to some of the students'.
EBE comment 5:	‘I think the sessions help medic students to understand the long‐term eventualities of people with brain injuries better. I also find a sense of satisfaction in knowing I potentially helped make some medic students better doctors in future. For example, a way in which I felt I was useful in the last session was when I described my situation in hospital: (paraphrasing) – ‘I was bleeding alot from everywhere and in order to reduce this the doctors gave me an agent that thickened my blood. It then turned out that because of the thickening agent I developed a blood clot which ended up starving my brain of oxygen for a period of time.’ This example of mine then led on to an interaction between a student and me, followed by a short talk from the CTF on how a doctor should approach decisions like this—such as the decision to thicken my blood, which was obviously the correct decision, but the eventualities might lead a student to think it was the wrong decision.’
EBE comment 6:	‘To give something back to the NHS. I feel useful helping the students to understand the effects of brain injuries on the survivors and their families. I feel satisfied when I'm contributing to their knowledge’.
EBE comment 7:	‘I like it feels like I've accomplished something for medical. To help new doctors understand TBI through a living organ not just a text book.’
EBE comment 8:	‘When the sessions aren't well facilitated some EBEs wonder whether they are more about a box ticking exercise rather than something useful.’

### 3.5 Section  3: Levels of Patient Engagement

The toolkit recognises that patient engagement exists on a continuum, ranging from minimal involvement (Level 1) to full partnership (Level 6) [3]. Section 3 provides an overview of these levels, drawing on established frameworks within the literature and encourages educators to carefully consider the appropriate level of engagement for their EBEs. Decisions about the level of involvement must account for support structures, institutional resources, patient availability and logistical considerations. A structured understanding of engagement levels facilitates systematic planning and enables gradual increases in patient involvement over time. Another important consideration should be financial remuneration. While not explicitly raised by the Silverliners group, the toolkit acknowledges that future expectations for compensating EBEs may grow, particularly considering current economic pressures. Institutions must consider the implications for budgets and the sustainability of EBE participation.

### 3.6 Section  4: Planning, Before You Start

Planning is critical to successful EBE integration. Section 4 provides a stepwise, structured approach for preparing EBE sessions. Each step offers practical guidance and links to relevant sections within the toolkit for further support. This preparatory framework ensures that sessions are purposefully designed, appropriately resourced and considerate of both educator and patient needs.

### 3.7 Sections 5 and 6: Curriculum Alignment

Aligning EBE sessions with the curriculum is essential to ensure that they complement learning objectives rather than functioning as ad hoc or tokenistic exercises [11, 12]. EBEs themselves have expressed concerns regarding misalignment and its potential to undermine the value of their participation (Table 2, EBE comment 8). Sections 5 and 6 offer guidance on identifying learning objectives, mapping sessions to the curriculum, integrating content sequentially and aligning assessments with session outcomes. The toolkit also highlights the value of embedding EBE sessions within multidisciplinary contexts, thereby enhancing the relevance and impact of patient contributions across the learning experience.

### 3.8 Sections 7 and 8: Recruiting Suitable EBEs

The selection of EBEs is critical to session success. EBEs emphasised the importance of recognising themselves as experts in their own experiences, offering essential insights into the patient journey and clinical decision‐making (Table 2, EBE comment 5). Sections 7 and 8 provide guidance on recruiting individuals whose narratives align with learning objectives while ensuring diversity in health conditions, cultural backgrounds and life experiences. Key considerations include the relevance of the medical condition, patient knowledge and expertise, willingness to share, availability and cultural sensitivity. Section 8 offers practical strategies for recruiting and sustaining a diverse group of EBEs, including lists of websites and networks that can facilitate access to varied patient populations.

### 3.9 Section 9: Confidentiality and Informed Consent

Some patients reported apprehension about discussing sensitive issues without clear safeguards. Existing practices, such as staff briefings or postsession evaluations, are often inconsistently applied and may lack patient‐centred focus [13]. Section 9 addresses these gaps by incorporating robust ethical guidelines, including informed consent procedures, confidentiality protocols and recommendations for establishing clear boundaries. These measures ensure that sessions protect patient well‐being, respect autonomy, and maintain the integrity and meaningfulness of contributions.

### 3.10 Section 10: Examples of Methods and Approaches

EBEs highlighted practical challenges that can impede participation, including physical accessibility, scheduling conflicts, fatigue and the repetitive nature of sharing emotionally taxing narratives (Table 2, EBE comments 2,3) [14]. The toolkit addresses these concerns by recommending flexible session formats, such as face‐to‐face, remote or prerecorded participation. Contingency plans, including ready‐to‐use recordings, are suggested to ensure session continuity. Section 10 includes examples from different medical schools, discussing the advantages and limitations of various approaches, thereby helping educators select the most appropriate format for their context.

### 3.11 Sections 11 and 12: Training and Orientation

Structured sessions with clear objectives and competent facilitation were valued highly by EBEs (Table 2, EBE comment 4). Sections 11 and 12 provide guidance on presession orientation for patients and training for facilitators. Best practice recommendations include allowing sufficient time for patient narratives, structuring opportunities for dialogue and ensuring that student questions are respectful and focused. Facilitator training is emphasised to ensure that sensitive discussions are managed appropriately and that constructive engagement with students is fostered. The toolkit provides a structured planning framework intended to support the design of EBE sessions that are meaningful and beneficial for participants.

### 3.12 Section 13: Evaluating Effectiveness

Finally, EBEs stressed the importance of evaluating whether their involvement genuinely enhances student learning rather than functioning as a checklist exercise. Section 13 outlines methods to measure the effectiveness of EBE participation, reinforcing the centrality of patient perspectives in shaping medical education. Suggested evaluation approaches include summative exam questions, surveys, reflective journals, OSCE integration and peer assessments. The toolkit also highlights the importance of longitudinal measures, such as revisiting empathy scores months postsession, to assess sustained impact on student development.

#### 3.12.1 Integrating Feedback Into the Toolkit

The toolkit provides a structured, evidence‐informed and patient‐centred resource for integrating EBEs into medical education. Each toolkit section corresponds to EBE‐derived themes. Introductory sections clarify the purpose of EBE sessions and their dual benefits. Planning sections focus on accessibility, logistics and emotional safety. Facilitator training, session evaluation and orientation modules are included to enhance quality and consistency. Patient voices guide content selection and delivery, supporting meaningful coproduction rather than tokenistic inclusion.

The EBE feedback was provided by DR (coauthor) from *The Silverlining Brain Injury Charity* and reflects both his perspective and that of the EBE group's broader engagement with the Toolkit. Overall, the feedback was positive, recognising the Toolkit's usefulness, clarity of themes and practical value for educators. However, several constructive points were raised to refine clarity and applicability

- **Structure and Clarity:** The EBEs initially found the document's structure confusing, suggesting clearer signposting (e.g., renaming the document to ‘A Toolkit to Enhance Early Medical Education Through Expert Patient Involvement’) and reordering sections to place an Introduction at the start.
- **Core Focus:** The reviewer identified Section 5 as the Toolkit's core and recommended reorganising earlier sections to make this clearer
- **Session Planning:** Emphasised that flexible session formats (e.g., recordings) require significant advance planning and recommended including contingency options for EBE absences
- **Financial Remuneration:** Highlighted the need to acknowledge the issue of payment for EBEs' time and contribution, noting that while participation is often altruistic, financial recognition may become increasingly important.
- **Selection Criteria:** Suggested rewording the ‘Experience and Knowledge,’ ‘Communication Skills’ and ‘Availability and Reliability’ criteria in Section 8 to reflect the diversity of EBE abilities—especially communication challenges due to conditions like aphasia—while valuing their insights.
- **Collaboration:** Recommended explicitly stating that collaboration includes partnerships with patient advocacy organisations and condition‐specific charities (e.g., The Silverlining).
- **Training and Preparation:** Noted that EBEs may not always receive formal training prior to participation and encouraged reflection on how best to support them
- **Appreciation and Recognition:** Found Section 13 (on recognising EBE contributions) particularly strong and valuable

This feedback‐driven model aligns with literature advocating for coproduction in public services and healthcare education [15, 16]. While some studies use larger samples, ours prioritised in‐depth insights over scale, consistent with recommendations for developing user‐centred tools [9, 11]. While this initial stage involved six EBEs with substantial prior experience in educational roles, future work will aim to validate and refine the Toolkit through broader codesign processes involving a larger and more diverse range of EBEs, students and educators.

We created the toolkit in two formats: one as a webpage to share more broadly (using Adobe Express) and another as a PDF document that users can easily navigate for continued reference. Providing multiple formats ensures wider accessibility and allows more users to benefit from the resource.

In conclusion, this toolkit demonstrates how patient voices can shape curricula, teaching methods and institutional practices. Built through collaboration with experienced EBEs, the toolkit offers a structured, flexible and ethically grounded framework for embedding patient perspectives into education at Level 3 of the involvement spectrum [3].

*This toolkit demonstrates how patient voices can shape curricula, teaching methods and institutional practices*.

Key features include the following:

- Emphasis on EBE dignity, contribution, and recognition.
- Flexible delivery formats to accommodate participation needs
- Clear session planning, orientation and facilitation guidelines.
- Ethical safeguards to support emotional well‐being and autonomy
- Evaluation methods to assess educational impact.

Comparable frameworks such as the NHS ‘It's OK to Ask’ *Toolkit* (https://tinyurl.com/mt8hp7ke) and the *European Patients Forum Patient Empowerment Toolkit* (https://tinyurl.com/bd2793fe) have informed the conceptual underpinning of our work. However, unlike these broader patient engagement resources, our Toolkit focuses specifically on guiding educators in effectively integrating EBEs into early medical and health professions curricula. The toolkit's focus on Level 3 was intentional to ensure clarity and relevance. However, future iterations may support higher levels of engagement, including shared decision‐making and coleadership. Progressing to EBE‐led session design, delivery and assessment will require structured training and confidence‐building and represents an important future direction for involvement initiatives. Ultimately, this codesigned toolkit reflects a shift towards more inclusive, responsive and values‐based education. It highlights how embedding lived experience in the classroom not only improves student learning but also strengthens healthcare's ethical and human foundations.

Toolkit webpage: https://new.express.adobe.com/webpage/BCYUViFlLQa3p

## Author Contributions

**Jaimy Saif:** conceptualization, methodology, writing – original draft, writing – review and editing, data curation, visualization. **David Rogers:** writing – review and editing, data curation. **Claire Stocker:** supervision, writing – review and editing, conceptualization, methodology, data curation.

## Funding

No external funding was received for this study.

## Conflicts of Interest

The authors declare no conflicts of interest.

## Supporting information


**Data S1:** Supporting Information.

## Data Availability

Data sharing not applicable to this article as no datasets were generated or analysed during the current study.
