# Supplementary material for: Enhancing Early Medical Education Through Patient Engagement: Creation of a Toolkit Informed by Experts by Experience
Source: Clin Teach. 2025 Dec 18;23(1):e70328. doi: 10.1111/tct.70328 (PMC12715381; doi:10.1111/tct.70328)
Supplement: Supplementary file 1 — Data S1: Supporting Information. [file TCT-23-e70328-s001.pdf]

A toolkit to enhance early medical education through expert by experience engagement

Dr Jaimy Saif, David Rogers, Dr Claire Stocker

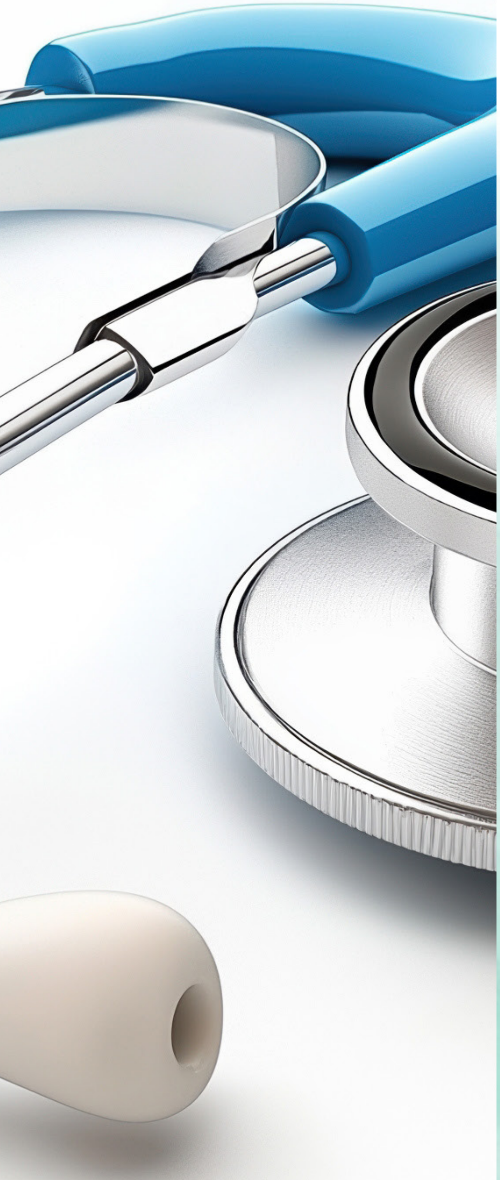

Table of contents

Section1: *What is an expert by experience (EBE) session?*
Section 2: *Benefits of EBE session*
Section 3: *Levels of patient engagement*
Section 4: *Planning before you start*
Section 5: *Aligning EBE session with the curriculum*
Section 6: *Interdepartmental collaborations*
Section 7: *Identifying suitable EBE*
Section 8: *Recruiting and maintaining diverse group of EBEs*
Section 9: *Ensuring confidentiality and consent*
Section 10: *Examples of approaches*
Section 11: *Pre-session orientation for students and enhancing student participation*
Section 12: *Training for EBEs*
Section 13: *Ways of evaluating the impact*

Before you begin..

Are you clear about why you need to involve expert by experience?

Refer to sections 1 and 2

⇓

Have you decided on what level of engagement you want from the expert by experience?

Refer to section 3

⇓

Have you effectively planned your expert by experience session?

Refer to sections 4,5,6

⇓

Have you decided which methods and approaches to use?

Refer to sections 10,11,12

⇓

Do you know how you will recruit your expert by experience?

Refer to sections 7, 8

⇓

Are you aware of the ethical considerations?

Refer to section 9

⇓

Do you know ways to evaluate the effectiveness of your expert by experience session?

Refer to section 13

Section 1: What is an expert by experience session

In the ever-evolving landscape of healthcare, it has become increasingly clear that medical education extends far beyond textbooks and lectures. To prepare future healthcare professionals for the challenges and complexities of patient care, a transformative approach has emerged- one that recognises the invaluable contributions of experts by experience. These individuals, who have first-hand experience living with specific medical conditions, offer a unique and essential perspective that textbooks alone cannot provide.

An expert by experience session is a learning experience where individuals with significant experience in managing their own chronic health conditions engage directly with students. During these sessions, patients share their personal stories, insights and how their illness affects their daily lives.

Section 2: Benefits of EBE session

Positive feedback from Aston Medical School

74%
Improved my understanding about the condition

66%
Improved my understanding about the clinical practice

Student feedback (n=135)

100%
Enjoyed the session

100%
Felt adequately supported

100%
Happy to be involved again

EBE feedback (n=13)

From literature (1-5), the involvement of EBEs in medical education offers several compelling benefits including:

- *Humanising healthcare*
- *Enhancing clinical skills*
- *Understanding patient perspective*
- *Fostering interdisciplinary collaboration*
- *Promoting cultural competence*
- *Encouraging reflective practice*
- *Supporting Evidence-Based Practice*
- *Inspiring advocacy and research*

Positive feedback from Aston Medical School

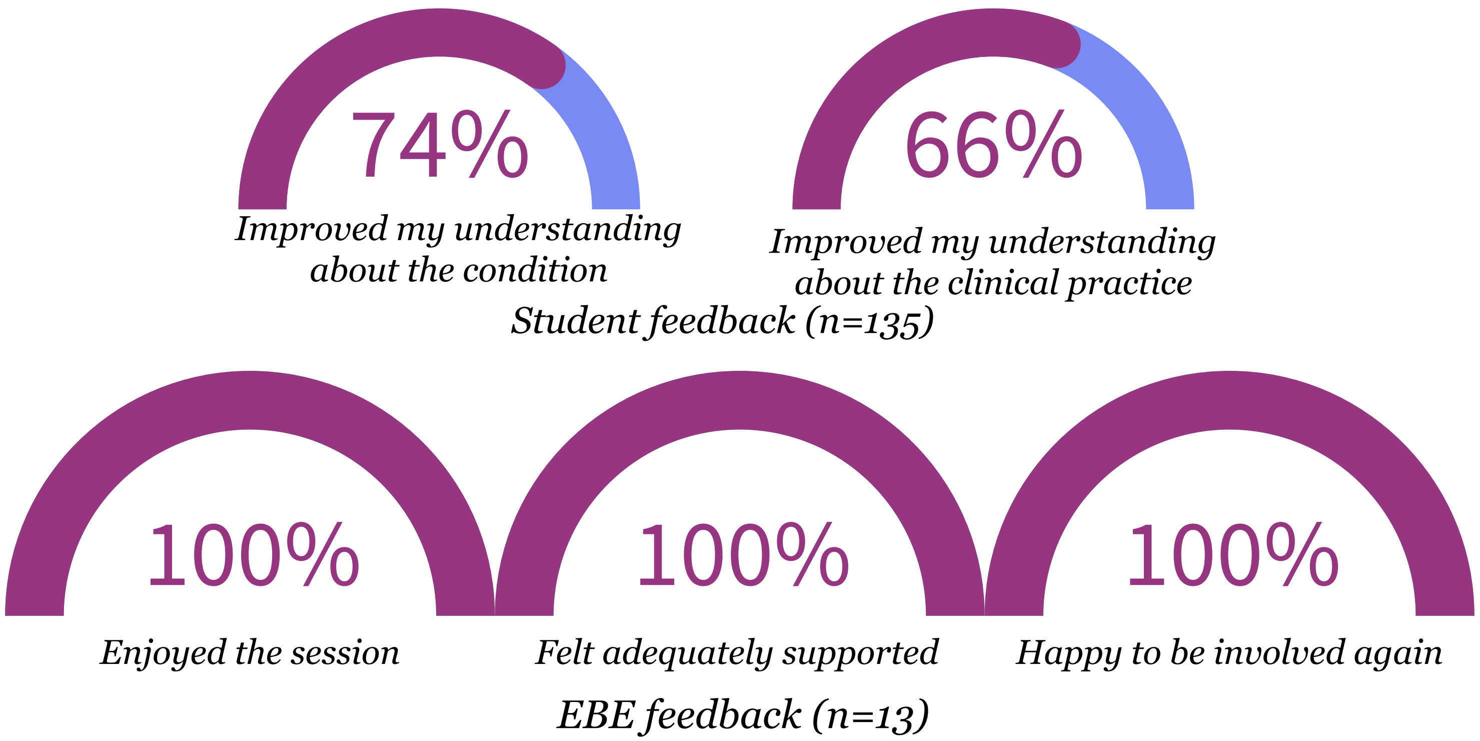

Section 3: Levels of patient engagement

Patients can be involved in medical education in numerous ways and to varying extents. Tools like the Cambridge Framework(6) and the Ladder of Involvement (7)are commonly used to evaluate the level of patient participation. Drawing from these models, a new approach, the Spectrum of Involvement (8) was developed to outline a continuum of patient engagement in medical education. This toolkit is focused on implementation at level 3.

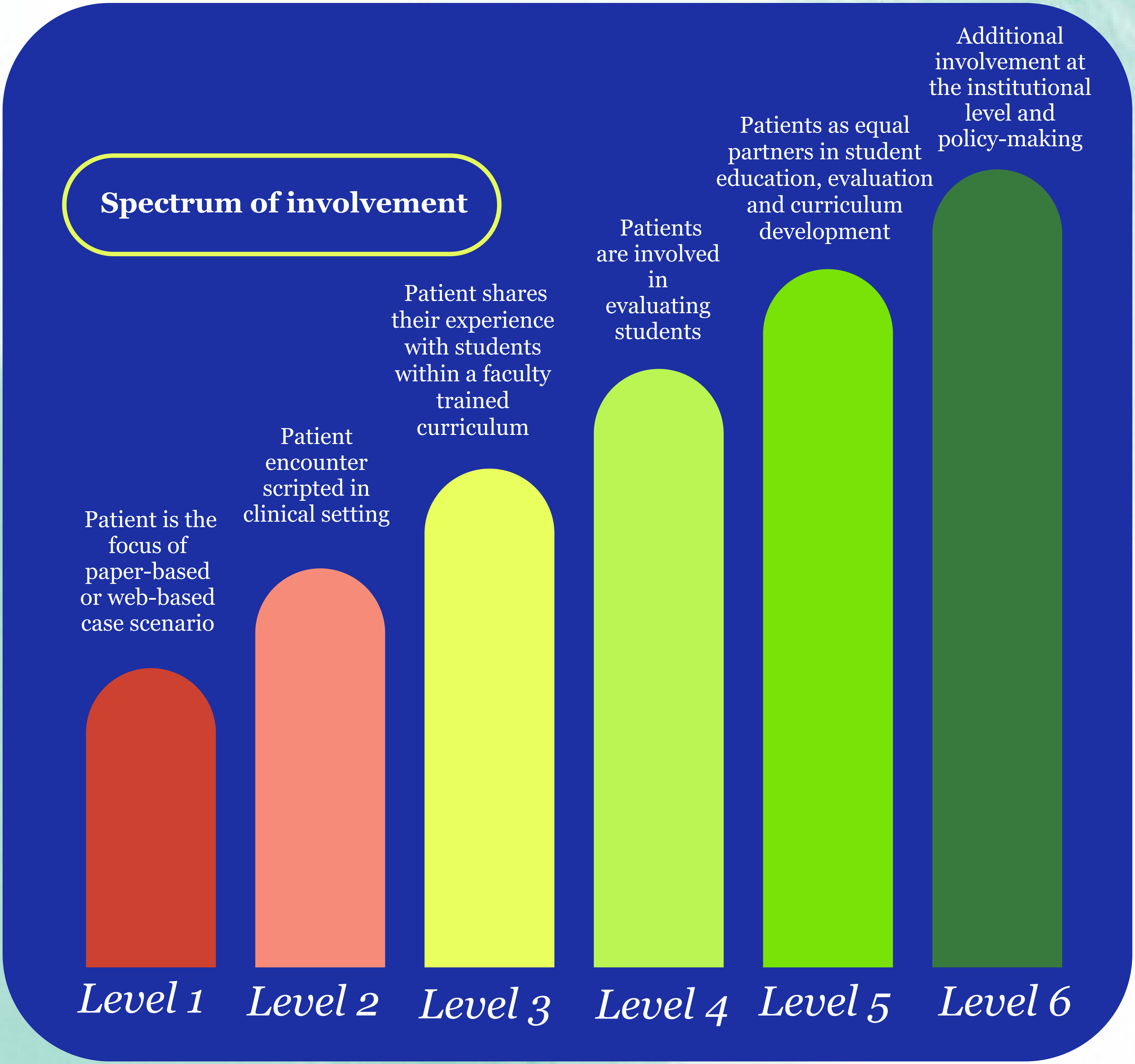

Section 4: Planning before you start

1. Define clear learning objectives and align EBE session within the curriculum. For some suggestions, please refer to section 5 of this toolkit.
2. Check for other related healthcare departments in the University for potential interdepartmental collaboration. For some suggestions, please refer to section 6 of this toolkit.
3. Select and recruit suitable patients: For some suggestions, please refer to section 7 and 8 of this toolkit.
4. Design the session structure: For some suggestions, please refer to section 10 of this toolkit.
5. Set up pre-session orientation for students: For some suggestions, please refer to section 11 of this toolkit.
6. Plan providing training and orientation of patients: For some suggestions, please refer to section 12 of this toolkit.
7. Organise logistics: Arrange the session location or online platform, ensuring accessibility for all participants. Ensure technical setup (microphones, video recording, etc.) is tested if the session will be recorded or live-streamed.
8. Gather and prepare evaluation materials: Create feedback surveys or questionnaires for students and EBEs to capture their thoughts on the session's effectiveness.
9. Identify ways to develop an evaluation method for assessing the session's impact on student understanding and empathy. For some suggestions, please refer to section 13 of this toolkit.

Section 5: Aligning EBE sessions with the curriculum

Aligning EBE sessions with the curriculum is crucial to ensure that these sessions complement and enhance the learning objectives and content covered in the program (9, 10). Here are some key points on how to achieve this alignment.

1. ***Identify learning objectives:*** *Determine what you want students to gain from these interactions. Ensure that these objectives align with the overall curriculum goals.*
2. ***Curriculum mapping:*** *Map out the existing curriculum to identify where EBE sessions can fit seamlessly.*
3. ***Integration into core topics:*** *Embed EBE sessions into core topics or themes covered in the curriculum. Match the expertise and experiences of the patients with the relevant subject matter.*
4. ***Sequential integration:*** *Introduce them at points in the curriculum where students have sufficient background knowledge to engage meaningfully and when the content aligns with the patient's experiences.*
5. ***Design session activities:*** *Develop specific session activities that challenge students to apply their clinical knowledge while considering the patient's perspective.*
6. ***Assessment alignment:*** *Ensure that assessments, reflect the content covered in expert patient sessions. Include questions or tasks that require students to integrate patient perspectives into their responses.*

Section 6: Interdepartmental collaborations for EBE session delivery

Integrating EBE sessions into a multidisciplinary setting, such as medicine, nursing, pharmacy, optometry, and physician associate programs, can greatly enhance the learning experience for students and improve patient care outcomes(11). Some strategies are:

Identify relevant topics: *Collaborate with faculty across disciplines to pinpoint pertinent topics benefiting from patient insights, such as chronic disease management, medication adherence, lifestyle adjustments, patient education, and healthcare system navigation.*

Recruit diverse EBEs: *Engage with patient advocacy groups, support organisations, and community networks to recruit a diverse range of expert patients eager to share their experiences, ensuring representation across various backgrounds, ages, ethnicities, and medical conditions for a comprehensive perspective.*

Develop structured sessions: *Design structured sessions that allow EBEs to interact with students from different backgrounds in a meaningful way. This could involve panel discussions, case studies, role-playing exercises, or small group activities focused on specific healthcare scenarios.*

Encourage interprofessional collaboration: *Emphasise the importance of interprofessional collaboration by including students from different disciplines in each session. Encourage students to work together to solve problems, exchange ideas, and learn from each other's perspectives.*

Provide preparatory materials: *Prior to the EBE sessions, provide students with preparatory materials such as background readings, videos, or online modules to familiarize them with the topic and prepare them for meaningful engagement with the expert patients.*

Facilitate open dialogue: *Create a supportive environment where students feel comfortable asking questions and engaging in open dialogue with patients. Encourage active listening, empathy, and respect for diverse viewpoints.*

Offer reflection opportunities: *Incorporate opportunities for students to reflect on their experiences interacting with EBEs. This could include structured debriefing sessions, written reflections, or group discussions to help students process their learning and insights gained from the sessions.*

Evaluate learning outcomes: *Implement pre- and post-session assessments to evaluate students' knowledge, attitudes, and skills related to patient-centered care and interprofessional collaboration. Use feedback from students and EBEs to continually improve the design and delivery of future sessions.*

Promote long-term engagement: *Encourage students to continue engaging with patients beyond the structured sessions by volunteering in community health initiatives, participating in patient support groups, or pursuing research projects focused on patient-centered care.*

Section 7: Identifying suitable EBEs

Relevant medical condition: *EBEs should have personal experience with a medical condition that is pertinent to the curriculum. Their condition should be one that students are likely to encounter in their future practice.*

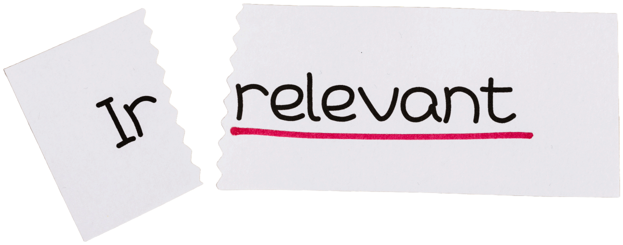

Experience and knowledge: *While some EBEs may have an in-depth understanding of their condition, others may offer more experiential or narrative insights. Both perspectives are valuable-whether grounded in clinical understanding, day-to-day challenges, or the emotional and social impact of living with the condition.*

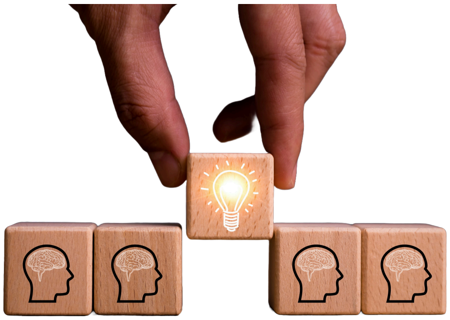

Communication: *EBEs should be able to share their story in a way that enables students to engage meaningfully with their experience. This may include non-verbal communication with the support of facilitators, particularly for EBEs with communication impairments (e.g., due to aphasia). These scenarios can enrich learning by highlighting real-world communication challenges in healthcare.*

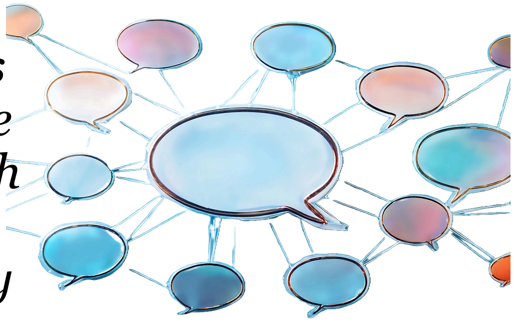

Willingness to share: *EBEs should be willing to discuss their medical journey, at a level that feels safe and appropriate for them.*

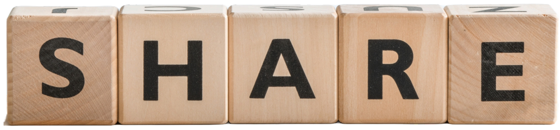

Availability: *Due to the nature of their health conditions, EBE availability may vary. It is often helpful to maintain a pool of EBEs and invite more than the minimum needed for a session, to account for last-minute changes.*

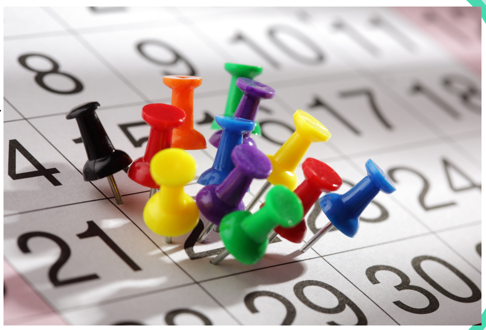

Remuneration and recognition*: EBEs should be appropriately acknowledged for their time and contribution. This may include financial remuneration, travel reimbursement, or other forms of recognition in line with institutional policy and the nature of their involvement.*

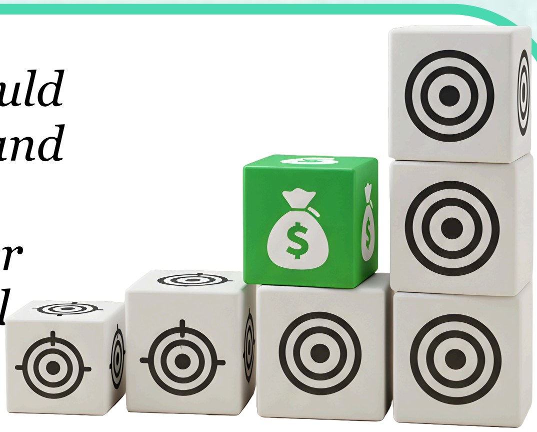

Cultural sensitivity: *To promote cultural competence, EBEs from diverse backgrounds may be preferred to provide students with exposure to different cultural perspectives on healthcare.*

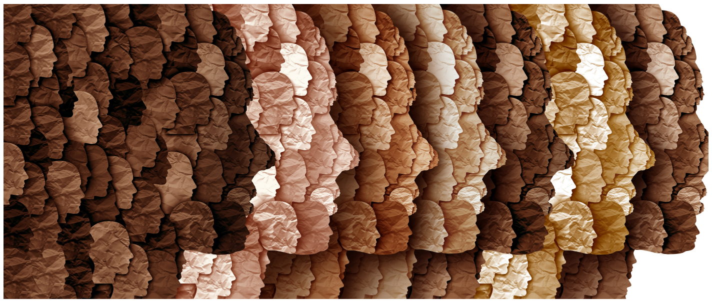

Section 8: Strategies for recruiting and maintaining diverse group of EBEs

Collaborate with patient advocacy and charity organisations: *These organisations often have networks of diverse individuals willing to share their experiences.*

www.thesilverlining.org.uk
www.patientslikeme.com
www.healthunlocked.com
www.healthtalk.org
www.patientvoices.org.uk
www.nationalvoices.org.uk
www.patients-association.org.uk

www.wego.health
www.rarepatientvoice.com
www.chronicdiseasecoalition.org
www.patientadvocate.org
www.patientworthy.com
www.rareconnect.org
www.creakyjoints.org

Community outreach: *Engage in community outreach programs to connect with potential EBEs. Attend support group meetings, health fairs, or community events to establish relationships with individuals who may be interested in participating*

Online forums and social media: *Utilise online patient forums, social media platforms, and healthcare-related websites to reach out to potential EBEs. These platforms often have active communities of individuals willing to share their experiences.*

Healthcare provider referrals: *Collaborate with healthcare providers who treat patients with diverse medical conditions. They can refer willing patients who are interested in contributing to medical education.*

Educational institutions: *Partner with universities, colleges, or schools that offer healthcare-related programs.*

Section 9: Ensuring confidentiality and informed consent

Strict privacy measures: *Implement strict privacy measures to safeguard the personal information of EBEs. Limit access to patient information only to individuals directly involved in the program.*

Use of anonymised data: *Whenever possible, use de-identified or anonymised data when discussing patient cases or experiences to protect the privacy of expert patients.*

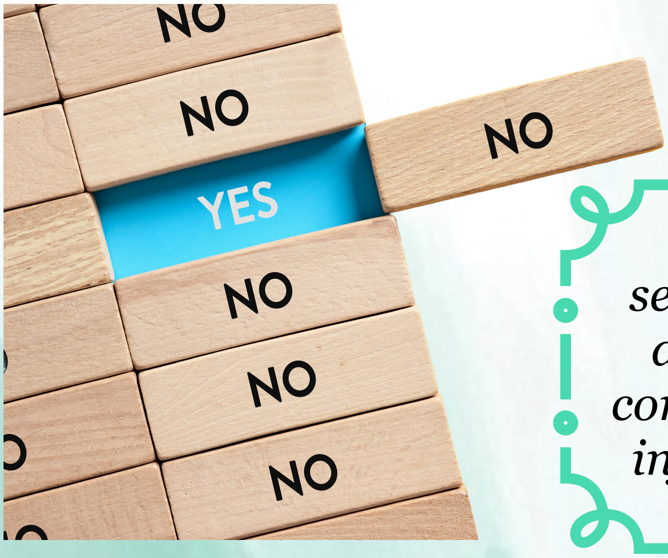

Secure communication: *Use secure communication methods such as encrypted email or secure video conferencing platforms, when sharing information related to EBE sessions.*

Clear explanation: *Provide EBEs with a clear and detailed explanation of the purpose, scope, and potential risks and benefits of their participation in medical education sessions.*

Consent documentation and ongoing consent: *Maintain written documentation of informed consent, including the date, the specific details of the consent process, and any signed consent forms. Recognise that informed consent is an ongoing process. Continuously communicate with EBEs, update them on any changes and obtain renewed consent if necessary.*

Section 10: Examples of methods and approaches

On-campus face to face sessions *Here the EBE is invited on-campus. They talk to the students in a lecture theatre or tutorial rooms for a fixed time and take questions from students at the end. This structure is followed in Aston Medical School. In the Year 1 and Year 2 of the medical program, EBE sessions are integrated into specific modules. These sessions feature EBEs who have conditions aligned with the core curriculum. They share their personal experiences and disease journeys with students, who have the chance to ask questions. Both patients and students receive briefing led by clinical teaching fellows prior to these sessions. There are also group sessions where a panel of EBEs collectively discuss their respective pathologies with the students.*

**Pros**	**Cons**
Ensure direct interaction with patients	Patient exhaustion
Engagement level of students is generally high due to patient presence	Not suitable for vulnerable patients such as those vulnerable to infections
Some patients report increased general satisfaction after being able to come to campus and meet students on-person	Not suitable for patients with spreadable disease
Easy for facilitators to facilitate	Can lead to last minute cancellations due to patient circumstances
Not affected by technology	Need close facilitation by clinical facilitators
	Scheduling and timetabling challenges

Section 10: Examples of methods and approaches

Remote sessions for interactive on-campus learning *In this setup, the EBE participates remotely, speaking to students who are physically present on campus. The session is streamed securely to the students, allowing them to engage with the patient in real-time. After sharing their experiences, the expert patient answers questions from the students, fostering an interactive learning environment. Aston Medical School also uses this structure for certain sessions, enhancing student engagement with patient perspectives in a hybrid format*

**Pros**	**Cons**
Suitable for vulnerable patients and most types of patients	Patient exhaustion
Less intimidating for EBEs involved compared to a face to face session	Can lead to last minute cancellations due to patient circumstances
Easier to organise compared to a face-to-face session	Need close facilitation by clinical facilitators
Easy for facilitators to facilitate	Scheduling and timetabling challenges
	Can be affected by technology

Aston Medical School
BIRMINGHAM UK

Section 10: Examples of methods and approaches

Pre-recorded sessions *In this approach, patients record narratives detailing their medical journeys, providing students with a rich resource that can be accessed on their own schedule. Bristol Medical School utilises pre-recorded patient narratives. They offer the "Patient and Public Involvement" module, where medical students have the opportunity to engage with pre-recorded patient stories(14). Following the recordings, students participate in live discussions or Q&A sessions, where they can interact with either the patients or faculty to deepen their understanding of the patient journey.*

**Pros**	**Cons**
Suitable for vulnerable patients and most types of patients	Need close facilitation by clinical facilitators
Prevent patient exhaustion	Scheduling and timetabling challenges
Easy for facilitators to facilitate	Can be affected by technology
Easier to organise compared to a face-to-face session	Students might not engage especially if they have not watched the patient story before hand
Less impacted by last minute cancellation due to patient circumstances	

University of BRISTOL

Bristol Medical School Colleges

Section 10: Examples of methods and approaches

Using interactive e-learning platforms *Patients and carers can engage with students through online discussion groups or serve as e-based consultants for problem-based learning. They may also create videos, written pieces, or develop discussion questions on specific issues. Online platforms are available for video interviews and personal stories, which can be used in educational settings, including interactive e-learning materials with virtual patients (few examples given in next page). University College London (UCL) Medical School uses e-learning resources such as the "Speaking Clinically" video archive, where patients discuss their medical conditions(15). This resource, available to Years 4 to 6, is provided by the Medical Schools Council and accessible to member institutions.*

Pros	Cons
Opportunity for co-creation from students, EBEs and academics	Require wrap up sessions consolidating learning outcomes
Prevent patient exhaustion	Students might not engage with the platform
Flexibility and creativity in patient stories	
Recording can be released when the relevant topic is taught	
Less impacted by last minute cancellation due to patient circumstances	

UCL MEDICAL SCHOOL

UCL

Section 10: Examples of online platforms with patient stories

- https://www.patientvoices.org.uk/find.htm
- www.thebody.com
- https://www.nlm.nih.gov/archive/20120918/hmd/breath/breathhome.html (click on 'interactives')
- www.nhs.uk/video/Pages/medialibrary.aspx?Tag=Real%20stories
- https://www.diabetes.org.uk/your-stories
- https://www.britishpainsociety.org/people-with-pain/patient-voices-video-resources/
- https://www.patients-association.org.uk/the-untold-patient-stories-shedding-light-on-the-realities-of-life-with-a-long-term-condition
- https://www.patients-association.org.uk/blog/how-to-use-patients-experience-of-care-to-improve-services
- https://storiesforchange.net/

Section 10: Examples of methods and approaches

Visits *In the Health in the Community module at Warwick Medical School (Year 1, Semester 1), students alternate weekly between classroom sessions and community-based learning. They regularly visit EBEs in local medical centres or their homes, exploring the local area and engaging with both the patient and their support network. These interactions provide insight into formal and informal caregiving and the social determinants of health. Students work in groups to interview patients and key carers, present their findings, and receive feedback from the patients, who are recruited by community nurses and compensated for their time. The module helps students understand coordinated care, the physician's role, and how a patient's life course shapes health outcomes.*

Pros	Cons
Powerful impact of 1-to-1 interaction	Not suitable for large student cohort
Provide good understanding of 'lay perspective' on health	Not suitable for vulnerable patients
Effective teaching of psychosocial aspects	Scheduling and timetabling challenges
Recording can be released when the relevant topic is taught	Require wrap-up sessions consolidating learning outcomes

WARWICK MEDICAL SCHOOL

Section 11: Ways to provide pre-session orientation and enhancing student participation

Designing engaging and interactive EBE sessions is essential for enriching medical education and increasing the impact and value of these sessions. Here are some key areas to consider.

1. ***Provide pre-session preparation:*** *Assign pre-session readings, videos, or activities to prepare students for the EBE session. This primes students with background knowledge and allows them to come prepared with thoughtful questions and reflections.*
2. ***Start with the patient story:*** *To Kick off the session, start with a patient story that highlights the topic being discussed. Recognising that some EBEs may find it challenging to recount their stories repeatedly, consider pre-recording the patient narratives at their convenience. These recordings can then be distributed to students prior to the session, enriching live Q&A discussions with more informed and stimulating interactions.*
3. ***Utilise different storytelling techniques:*** *Incorporate a variety of storytelling techniques to keep students engaged and interested. This could include personal narratives, testimonials, case studies, role-playing scenarios, and interactive storytelling activities. Incorporating multimedia elements such as videos, photos and audio recordings can all bring patient experiences to life. These can all aid in evoking emotions, capturing attention and deepen understanding for students.*

Section 11: Ways to provide pre-session orientation and enhancing student participation

4. Connect storytelling to learning objective: *Ensure that storytelling is integrated seamlessly with educational content and aligned with learning objectives. Use patient stories to illustrate key concepts, reinforce learning points, and stimulate critical thinking and reflection.*

5. Provide contextual information: *Supplement storytelling with relevant contextual information, background knowledge, and educational content. Help students understand the broader context of the patient's story, including medical terminology, disease processes, treatment options, and healthcare system factors.*

6. Facilitate dialogue: *Foster open dialogue and discussion between EBEs and students. Encourage students to ask questions, share their thoughts and reactions, and engage in meaningful conversations with EBEs about their experiences.*

7. Facilitate reflection: *Build in time for reflection and debriefing at the end of the session. Encourage students to reflect on the patient stories they've heard, consider the implications for their future practice, and identify lessons learned that they could apply in clinical settings.*

8. Timely feedback: *Gather feedback from both students and EBEs about the storytelling experience. Use feedback to evaluate the effectiveness of the session, identify strengths and areas for improvement, and adjust future sessions accordingly.*

Section 12: Training and orientation guidelines for EBEs

Training and orientation guidelines for EBEs play a vital role in optimising their contributions to EBE sessions in medical schools, ultimately benefiting both patients and students and advancing the goals of patient-centered medical education. Here are some key areas to consider.

1.Orientation session: *Hold an initial orientation session to introduce EBEs to the goals, format, and expectations of the EBE sessions. Provide an overview of the medical school curriculum, the role of EBEs in the educational process, and the importance of patient-centered care.*

2.Training modules: *Develop training modules covering topics such as effective communication skills, confidentiality and privacy regulations, understanding the student perspective and providing constructive feedback. These can be developed by collaborating with Learning Development Centre within the University. These modules can be delivered in-person or through online resources.*

3.Feedback mechanisms: *Establish feedback mechanisms for EBEs to provide input on their experiences participating in the sessions. Encourage them to share suggestions for improvement and areas where they feel additional support or training is needed.*

4.Cultural sensitivity training: *Offer cultural sensitivity training to help EBEs navigate diverse student populations and ensure their experiences are understood and respected by students from different backgrounds.*

Section 12: Training and orientation guidelines for EBEs

5. Supportive environment: *Foster a supportive environment where EBEs feel valued, respected, and empowered to contribute to the educational process. Arrange events to recognise and celebrate their contributions to medical education.*

6. Respect for confidentiality: *Emphasise the importance of confidentiality and privacy when sharing personal health information or experiences. Clearly communicate the boundaries around what information can be shared during the sessions and how it should be handled.*

7. Consent process: *Implement a robust consent process that outlines how EBEs' information will be used and shared during the sessions. Ensure that EBEs understand and consent to their participation, including any recording or dissemination of session materials.*

8. Boundaries on advice giving: *Clarify that EBEs are not expected to provide medical advice or treatment recommendations to students. Encourage them to share their experiences and insights while emphasising that medical decision-making remains the responsibility of healthcare professionals.*

9. Self-care and well-being: *Encourage EBEs to prioritise their own well-being and self-care throughout their involvement in the sessions. Provide resources and support for managing emotional or psychological challenges that may arise from sharing personal experiences.*

Section 13: Ways of evaluating the impact of EBE sessions

It is important to evaluate the impact of the session on student understanding and empathy. Below are some reported ways from the literature.

1.Summative exam questions: *Develop questions in summative exams focused on scenarios involving empathy and patient understanding, which allow students to apply learning from EBE sessions (16).*

2. Pre- and post-session surveys: *Implement pre- and post-session surveys measuring changes in empathy using validated tools such as the Jefferson Scale of Empathy. This helps to compare attitudes before and after interaction with EBEs (17).*

3. Reflective journals: *Require students to write reflective journals post-session, focusing on how the experience influenced their perspective on patient care and empathy. Using reflective practice has been shown to deepen empathy(18).*

4. OSCE incorporation: *Incorporate aspects of patient empathy in Objective Structured Clinical Examinations (OSCEs), where students interact with standardised patients to gauge empathy and interpersonal skills in a controlled environment (19).*

5. Peer assessments: *Use peer evaluations where students assess each other's engagement and sensitivity during group discussions on patient experiences. This approach fosters accountability and reflection, as noted by the General Medical Council (https://www.gmc-uk.org/).*

6. Longitudinal follow-up: *Track changes in empathy over time by revisiting empathy scores and reflections several months post-session (16).*

References

1. Stephens E, William L, Lim L-L, Allen J, Zappa B, Newnham E, et al. Complex conversations in a healthcare setting: experiences from an interprofessional workshop on clinician-patient communication skills. BMC Medical Education. 2021;21(1):343.
2. Kaiser L, Conrad S, Neugebauer EAM, Pietsch B, Pieper D. Interprofessional collaboration and patient-reported outcomes in inpatient care: a systematic review. Systematic Reviews. 2022;11(1):169.
3. Rukadikar C, Mali S, Bajpai R, Rukadikar A, Singh AK. A review on cultural competency in medical education. Journal of family medicine and primary care. 2022;11(8):4319-29.
4. Ottewill R, Demain S, Ellis-Hill C, Greenyer CH, Kileff JJMt. An expert patient-led approach to learning and teaching: the case of physiotherapy. 2006;28(4):e120-e6.
5. Aguayo-González M, San Rafael-Gutiérrez S, Gómez-Ibáñez R, Leyva-Moral JMJIJoNES. Narrative photography with an expert patient as a method to improve empathy: A satisfaction study with health sciences students. 2022;19(1):20210124.
6. Spencer J, Blackmore D, Heard S, McCrorie P, McHaffie D, Scherpbier A, et al. Patient‐oriented learning: a review of the role of the patient in the education of medical students. 2000;34(10):851-7.
7. Tew J, Gell C, Foster S. Learning from Experience: Involving service users and carers in mental health education. 2004.
8. Towle A, Bainbridge L, Godolphin W, Katz A, Kline C, Lown B, et al. Active patient involvement in the education of health professionals. 2010;44(1):64-74.
9. Balzer F, Hautz WE, Spies C, Bietenbeck A, Dittmar M, Sugiharto F, et al. Development and alignment of undergraduate medical curricula in a web-based, dynamic Learning Opportunities, Objectives and Outcome Platform (LOOOP). 2016;38(4):369-77.
10. Mahzari M, AlNahedh T, Ahmed AA, Al Rumyyan A, Shaban S, Magzoub MEJAiME, et al. Practical guide to undergraduate medical curriculum alignment and mapping. 2023:1001-12.
11. Blickem C, Priyadharshini EJJoIC. Patient narratives: The potential for "patient-centred" interprofessional learning? 2007;21(6):619-32.
12. Chan KKC, Samoutis G. Patient-centered medicine: The use of expert patients in medical education. 2020.
13. Bazzano LA, Durant J, Brantley PRJOJ. A modern history of informed consent and the role of key information. 2021;21(1):81-5.
14. Dost S, Hossain A, Shehab M, Abdelwahed A, Al-Nusair LJBo. Perceptions of medical students towards online teaching during the COVID-19 pandemic: a national cross-sectional survey of 2721 UK medical students. 2020;10(11):e042378.
15. UCL. UCL [Available from: www.ucl.ac.uk/medical-school/current-mbbs-students/e-learning-guidance.
16. Winter R, Issa E, Roberts N, Norman RI, Howick JJBo. Assessing the effect of empathy-enhancing interventions in health education and training: a systematic review of randomised controlled trials. 2020;10(9):e036471.
17. Ferri P, Rovesti S, Padula MS, D'Amico R, Di Lorenzo RJPR, Management B. Effect of expert-patient teaching on empathy in nursing students: a randomized controlled trial. 2019:457-67.
18. DasGupta S, Charon RJAM. Personal illness narratives: using reflective writing to teach empathy. 2004;79(4):351-6.
19. Dufayet L, Piot M-A, Geoffroy P-A, Oulès B, Petitjean-Brichant C, Peiffer-Smadja N, et al. CARECOS study: Medical students' empathy as assessed with the CARE measure by examiners versus standardized patients during a formative Objective and Structured Clinical Examination (OSCE) station. Medical Teacher. 2024;46(9):1187-95.

Acknowledgements

We thank the experts by experience from Silverlining Brain Injury Charity for their time and valuable feedback.
